# Screening Ultrasonography in Children with Prolonged Fever Can Detect Early Takayasu Arteritis

**DOI:** 10.31662/jmaj.2021-0160

**Published:** 2021-10-01

**Authors:** Sathish Kumar, Sridhar Gibikote, Debashish Danda

**Affiliations:** 1 Departments of Pediatric Rheumatology, Radio Diagnosis and Clinical Immunology & Rheumatology, Christian Medical College Hospital, Vellore, India

**Keywords:** Takayasu arteritis, children, ultrasound, fever of unknown origin

Takayasu arteritis (TAK) is a rare disease. Takayasu arteritis in children (c-TAK) is even rarer (2 per million population). However, c-TAK has some unique distinctions compared to adult-onset TAK. One of the unique features of c-TAK includes fever of unknown origin (FUO) as a presenting feature in 80% of cases; however, c-TAK constitutes less than 5% of FUO in children. Ultrasonography (USG) is a first-line evaluation procedure for FUO in children. In a case series and review of literature by Hisataka Nozawa et al. in this issue, the authors discuss the utility of USG in the diagnosis of early c-TAK and present their small retrospective series of children with c-TAK who had presented with FUO.

Undoubtedly, this series does not have the power to generalize its observations. However, the challenge of diagnosing early c-TAK mandates the exploration of existing data to identify an early therapeutic window of opportunity and that depends on early diagnosis to achieve an improved outcome. c-TAK has a paucity of specific symptoms and laboratory biomarkers, which makes the disease often unrecognized at onset, and its activity is frequently underestimated. Constitutional symptoms, including fever, arthralgia, weight loss, headache, abdominal pain, and elevated acute phase reactants, are characteristic features of the pre-pulseless (stenosis) phase of TA ^(1)^. Only 33% of patients with TA are diagnosed in this early phase, despite the advances in imaging techniques and awareness about the disease in the last decade ^(2)^.

Pediatricians face the biggest challenge in diagnosing c-TAK in the pre-pulseless phase for the reasons described above; tissue biopsy is also not a feasible option to pick up early c-TAK. Imaging techniques, therefore, still remain the most important diagnostic tools used in suspected c-TAK in the pre-stenotic phase. Thus, diagnosis of c-TAK is usually confirmed by imaging modalities, such as CT, PET-CT, or MR angiogram, but the use of contrast, invasive nature of these procedures, and movement by small children within CT or MRI tunnels can be limiting factors in children. In the light of these issues, early pre-stenotic disease may be preferably detected by noninvasive ultrasound because of its high resolution and excellent depiction of arterial walls ^(3)^. In suspected c-TAK, the carotid, subclavian, renal, and femoral arteries should be examined along with the abdominal aorta in patients with a lean body mass. This is where the advantage of USG lies in visualizing large vessels of the abdomen, because of less abdominal fat and gas shadows in children than in adults.

Recent studies also indicate that contrast-enhanced ultrasonography is a potential imaging modality for diagnosis and monitoring disease activity in TAK, which may be even more sensitive than the laboratory indicators ^(4)^. However, contrast is again a limiting factor in children.

Although this small sample size and retrospective data cannot contribute to the calculation of sensitivity or specificity of USG as a diagnostic tool in c-TAK, this piece of information adds invaluable data to the literature on this subject. In this series of five children with c-TAK described by these authors, USG was reported to detect abnormal arteries in the abdomen and neck regions, with a moderate concordance rate compared to CECT ^(5)^. These five children with c-TAK were detected among the pediatric FUO cohort in a referral center over a 14-year period using USG, possibly reflecting a real-world scenario; this is because c-TAK predominantly presents as FUO and USG was used as the first-line evaluation in their cohort. In fact, USG is an often used modality in the workup of FUO in children ^(6)^. Hence, ultrasound can be used as a screening test in the diagnosis of c-TAK in the pre-stenotic phase. Implications of early diagnosis of c-TAK in the pre-stenotic stage include timely immunosuppressive treatment to improve outcomes of c-TAK, as reported in this series.

Ultrasound visualization of the origin of the main branches of the aorta can sometimes be impossible and time consuming. However, if such an attempt is made in children, especially in clinical scenario like FUO, it may upscale the pretest probability of diagnosing early c-TAK. The higher prevalence of abdominal vessel involvement in c-TAK than in adult-onset TAK makes it an even more relevant procedure in c-TAK ^(7)^. Interestingly, in children, ultrasound may be superior to CECT for neck vessels also, according to this study, because the low fat in neck regions of children contributes to poor differentiation between the periarticular soft tissue and the arterial wall in CECT. The authors’ literature review also shows a scarcity of data on USG as an initial screening method to detect early c-TAK as well as early adult-onset TAK.

Despite limitations, USG as a noninvasive method is of immense value to detect early c-TAK. This retrospective series and review of literature generates a new research question for the diagnosis of early c-TAK by USG; the pooling of large, multicentric prospective data is needed.

## Article Information

### Conflicts of Interest

None
